# Predictive factors for sepsis by carbapenem resistant Gram-negative bacilli in adult critical patients in Rio de Janeiro: a case-case-control design in a prospective cohort study

**DOI:** 10.1186/s13756-020-00791-w

**Published:** 2020-08-14

**Authors:** Elisangela M. Lima, Patrícia A. Cid, Debora S. Beck, Luiz Henrique Z. Pinheiro, João Pedro S. Tonhá, Marcio Z. O. Alves, Newton D. Lourenço, Roberto Q. Santos, Marise D. Asensi, José Aurélio Marques, Carolina S. Bandeira, Caio Augusto S. Rodrigues, Saint Clair S. Gomes Junior, Marisa Z. R. Gomes, the Nucleus of Hospital Research study collaborators, Glória Regina A. A. Magalhães, Priscila P. Silva, Scyla Maria S. A. S. Reis Di Chiara, Amanda Aparecida S. Machado, Thaisa M. Tozo, Lucas Lameirão P. A. Rosas, Paulo C. M. Barros, Wania V. de Freitas, Ana Paula D. C. Assef

**Affiliations:** 1 Laboratório de Genética Molecular de Microrganismos, Instituto Oswaldo Cruz, Oswaldo Cruz Foundation (IOC/FIOCRUZ), Rio de Janeiro, RJ Brazil; 2 Laboratório de Pesquisa em Infecção Hospitalar, Instituto Oswaldo Cruz, Oswaldo Cruz Foundation (IOC/FIOCRUZ), Rio de Janeiro, RJ Brazil; 3 Hospital Federal dos Servidores do Estado (HFSE), Ministry of Health, Rio de Janeiro, RJ Brazil; 4 Instituto Fernandes Figueira, Oswaldo Cruz Foundation, Rio de Janeiro, RJ Brazil

**Keywords:** Sepsis, Gram-negative bacilli, Antimicrobial resistance, Risk factors, Hospital infection, Polymerase chain reaction

## Abstract

**Background:**

Studies have investigated risk factors for infections by specific species of carbapenem-resistant Gram-negative bacilli (CR-GNB), but few considered the group of GNB species and most of them were performed in the setting of bacteremia or hospital infection. This study was implemented to identify risk factors for sepsis by CR- and carbapenem-susceptible (CS) GNB in intensive care unit (ICU) patients to improve management strategies for CR-GNB sepsis.

**Methods:**

We developed a case-case-control study from a prospective cohort of patients with systemic inflammatory response syndrome (SIRS), sepsis-2 or sepsis-3 criteria in which blood and other sample cultures were collected and antimicrobial therapy was instituted, in an adult clinical-surgical ICU, at tertiary public hospital in Rio de Janeiro, from August 2015 through March 2017.

**Results:**

Among the total of 629 ICU admissions followed by 7797 patient-days, after applying inclusion and exclusion criteria we identified 184 patients who developed recurrent or single hospital-acquired sepsis. More than 90% of all evaluable cases of sepsis and 87% of control group fulfilled the modified sepsis-3 definition. Non-fermenting bacilli and ventilator-associated pneumonia predominated as etiology and source of CR-GNB sepsis. While Enterobacteriaceae and intra-abdominal surgical site plus urinary-tract infections prevailed in CS-GNB than CR-GNB sepsis. Carbapenemase production was estimated in 76% of CR-GNB isolates. Multivariate logistic regression analysis revealed previous infection (mostly hospital-acquired bacterial infection or sepsis) (OR = 4.28; 95% CI 1.77–10.35), mechanical ventilation (OR = 4.21; 95% CI 1.17–15.18), carbapenem use (OR = 3.42; 95% CI 1.37–8.52) and length of hospital stay (OR = 1.03; 95% CI 1.01–1.05) as independent risk factors for sepsis by CR-GNB. While ICU readmission (OR = 6.92; 95% CI 1.72–27.78) and nosocomial diarrhea (OR = 5.32; 95% CI 1.07–26.45) were factors associated with CS-GNB sepsis.

**Conclusions:**

The investigation of recurrent and not only bacteremic episodes of sepsis was the differential of this study. The results are in agreement with the basic information in the literature. This may help improve management strategies and future studies on sepsis by CR-GNB.

## Background

Sepsis is a leading cause of morbidity and mortality, during and after hospitalization in intensive care units (ICU) [1, 2]. Carbapenem-resistant (CR) *Acinetobacter baumannii*, *Pseudomonas aeruginosa* and Enterobacteriaceae have become major pathogens, especially in ICUs, implicated in healthcare-associated sepsis, causing prolonged hospitalization, high mortality, and increased costs [3–6].

Several studies in the literature have investigated risk factors for infections by specific species of CR-Gram-negative bacilli (GNB) [7–17], but few considered all detected GNB species [18, 19], and most of them were in the context of nosocomial infection [9, 10, 12, 15–17] or bacteremia [8, 11, 18, 19].

The empirical therapy of sepsis should be started within the first hour of presumed diagnosis, at a time when the clinic-epidemiological characteristics remain as the only determinants of a patient at greater risk.

Given these facts, we performed a case-case-control study to investigate predictive factors for sepsis by CR- and carbapenem-susceptible (CS) GNB in adult patients from a Brazilian public ICU. Our goal is to develop and validate a predictive score to identify patients at higher risk for CR-GNB sepsis in future studies.

## Methods

### Patients, setting and study design

The study followed a case-control design from a prospective cohort of patients with SIRS, sepsis-2 or sepsis-3 criteria in which blood and other samples’ cultures were collected and antimicrobial therapy was instituted, for two or more days, in an adult clinical-surgical ICU, at a tertiary public hospital in Rio de Janeiro, Brazil, from August 2015 through March 2017. This study was approved by the institutional ethics committee and followed the Declaration of Helsinki and its later amendments. The study followed the STROBE recommendations for observational cohort studies (STROBE list in Additional file 1: Appendix S4) [20].

We evaluated all patients` clinical and surveillance samples cultured during the episodes, throughout the ICU stay, and the follow-up period of 30 days following the end of sepsis treatment. Surveillance cultures were not used to assess patient inclusion or exclusion in the study. Patients were not matched by any variable, considering the homogeneity of this population.

We followed all detected sepsis episodes during the ICU stay and follow-up period. Patients with more than one episode were aleatory selected in each group in such a manner that patients who presented CR-GNB sepsis were selected as case 1, patients who had CS-GNB sepsis episode were selected as case 2 and those with unknown sepsis or due to other etiologies than GNB were selected as control group. Cases and controls entered the study once and were monitored closely during the follow-up period.

We excluded patients younger than 18 years old, those with sepsis acquired in the community or associated with another healthcare institution, those who refused to sign the consent form and those suffering from polymicrobial sepsis by GNB and non GNB agents. In addition, we excluded patients initially enrolled in the control or CS-GNB case group that evolved respectively with CS-GNB or CR-GNB infection after discharge from ICU and during the follow-up period.

The variables investigated as predictive factors were studied during the period of hospitalization prior to sepsis episode for both cases and controls. We investigated demographics and comorbidities, length of hospital stays, prior ICU hospitalization, reasons for ICU admission, simplified acute physiology *score* (SAPS-3) and sequential organ failure assessment (SOFA) at baseline, previous use of invasive devices and antimicrobials. The variables and their definitions are described in Tables 1 and 2. The information was collected from multiple sources including hospital records, hospital laboratory system, radiological records, hospital infection control committee daily surveillance, ICU staff daily clinical round records, and data entered daily into the ICU Epimed System. We collected and managed study data using REDCap electronic data capture tools hosted at Instituto Oswaldo Cruz, Fundação Oswaldo Cruz (IOC/FIOCRUZ). The study formularies with the investigated variables are in Additional file 1: Appendices S1, S2 and S3. To avoid potential bias, all data collected were standardized and monitored throughout the study.

**Table 1:** Clinical characteristic investigated for association with CR-GNB sepsis (Case group 1) in an adult clinical-surgical ICU

Predictive Factors	Univariate analysis		Odds ratio	95% CI	*P*^t^ valor
	CR-GNB (*n* = 60)	Controls (*n* = 94)			
Demographic data					
Age in years, median (range)	62 (23–91)	62 (19–92)	1.00	0.98–1.02	0.95
Male sex, n (%)	36 (60)	46 (49)	1.57	0.81–3.02	0.18
Comorbidities^a^, n (%)					
Diabetes mellitus^b^	21 (35)	35 (37)	0.91	0.46–1.78	0.78
Renal failure^c^	30 (50)	38 (40)	1.47	0.77–2.83	0.25
Hemodialysis^d^	22 (37)	18 (19)	2.44	1.17–5.10	0.02
Chronic liver disease^e^	2 (3)	9 (10)	0.33	0.07–1.56	0.16
Immunossupressive condition^f^	9 (15)	11 (12)	1.33	0.52–3.43	0.55
Gastrointestinal disease	16 (27)	18 (19)	1.54	0.71–3.31	0.27
Geniturinary disease	5 (8)	9 (10)	0.86	0.27–2.70	0.79
Pulmonary disease	10 (17)	14 (15)	1.14	0.47–2.77	0.77
AIDS or chronic infectious disease	3 (5)	3 (3)	1.60	0.31–8.18	0.56
Surgery^g^	26 (43)	46 (49)	0.80	0.42–1.53	0.50
Infection/Colonization by CR-GNB	19 (32)	7 (7)	5.76	2.24–14.79	< 0.001
Nosocomial diarrhea^h^	17 (28)	6 (6)	5,.80	2.13–15.75	0.001
Neutropenia^i^	5 (8)	4 (4)	2.05	0.53–7.94	0.30
Neoplasm	23 (38)	28 (30)	1.47	0.74–2.90	0.27
Infection	46 (77)	31 (33)	6.68	3.20–13.95	< 0.001
Prior ICU hospitalization, n (%)	7 (12)	5 (5)	2.35	0.71–7.78	0.26
Length of hospital stay (in days)					
Median (range)	26.5 (1–375)	10 (0–143)	1.04	1.02–1.06	< 0.001
ICU hospitalization reason, n (%)					
Elective or emergency surgery	10 (17)	28 (30)	0.47	0.21–1.06	0.07
Respiratory tract disease	16 (27)	14 (15)	2.08	0.93–4.65	0.08
Cardiovascular disease	6 (10)	8 (9)	1.19	0.39–3.63	0.75
Neurological disease	2 (3)	11 (12)	0.26	0.06–1.22	0.09
Gastrointestinal disease	3 (5)	5 (5)	0.94	0.22–4.07	0.93
Renal pathology	5 (8)	11 (12)	0.69	0.23–2.08	0.51
Sepsis	31 (52)	30 (32)	2.40	1.23–4.68	0.01
Sepsis shock	23 (38)	27 (39)	1.63	0.82–3.24	0.17
Total SOFA score^j^ at ICU admission,					
Median (range)	6 (1–17)	6 (0–17)	1.06	0.98–1.15	0.16
SAPs 3 score^k^, median (range)	65 (30–103)	64 (29–105)	1.01	0.99–1.03	0.39
Invasive devices, n (%)					
Mechanical ventilation	56 (93)	58 (62)	8.69	2.90–26.01	< 0.001
Central vascular catheter	60 (100)	81 (86)	...	...	0.003
Urinary catheter	55 (92)	72 (76)	3.16	1.12–8.92	0.03
Previous use of antimicrobials, n (%)					
Aminoglycosides^l^	18 (30)	7 (7)	5.33	2.07–13.74	0.001
Cephalosporins, 3rd and 4rd generations^m^	8 (13)	16 (17)	0.75	0.30–1.88	0.54
Carbapenems^n^	48 (80)	32 (34)	7.75	3.61–16.62	< 0.001
Glycopeptides^o^, linezolid and tigecycline	45 (75)	35 (37)	5.06	2.47–10.37	< 0.001
Fluoroquinolones^p^	14 (23)	15 (16)	1.60	0.71–3.62	0.26
Metronidazole	11 (18)	13 (14)	1.40	0.58–3.37	0.46
Piperacilin-tazobactam	30 (50)	21 (22)	3.48	1.72–7.01	< 0.001
Polymyxins^q^	22 (37)	9 (10)	5.47	2.30–12.98	< 0.001
ATB with action for anaerobes^r^	56 (93)	59 (63)	8.31	2.77–24.88	< 0.001
Antifungal agents^s^	26 (43)	13 (14)	4.77	2.19–10.36	< 0.001

*Abbreviations*: *AIDS* Acquired Immunodeficiency Syndrome, *ATB* antibiotic agents, *CI* Confidence interval, *CR-GNB* Carbapenem resistant Gram-negative bacilli, *ICU* Intensive Care Unit

^a^Prior comorbidities or conditions to investigated sepsis episode

^b^Diagnosis of diabetes mellitus requiring oral or injectable hypoglycemic drug

^c^Creatinine clearance < 30 cc/min

^d^Required in the last 90 days

^e^Laboratory clinical evidence

^f^ Prednisone > 10 mg for more than 50 days, corticosteroid for > 7 days or immunomodulatory agents (examples: monoclonal agents, methotrexate)

^g^In the last 30 days

^h^Nosocomial diarrhea (3 or more daily episodes of stool for 2 or more days)

^i^Granulocytes < 500 cells/mm^3^

^j^Sequential Organ Failure Assessment score

^k^Simplified Acute Physiology Score III

^l^Amikacin and gentamicin

^m^Ceftriaxone, ceftazidime and cefepime

^n^Ertapenem, imipenem-cilastatin and meropenem

^o^Daptomycin, teicoplanin and vancomycin

^p^Ciprofloxacin, levofloxacin, moxifloxacin

^q^Polymyxin B and colistin

^r^Antibacterial agents with action for anaerobes – Amoxicilin-clavulanate, ampicillin-sulbactam, piperacilin-tazobactam, clindamycin, ertapenem, imipenem, meropenem and metronidazole

^s^Amphotericin B family (standard, lipid complex or liposomal Amphotericin), echinocandins and azoles

^t^Pearson’s chi-square test or Fisher’s exact test or Mann-Whitney-Wilcoxon U test, as required, and considering statistically significant *p* < 0.05

**Table 2:** Clinical characteristic investigated for association with CS-GNB sepsis (Case group 2) in an adult clinical-surgical ICU

Predictive Factors	Univariate analysis		Odds ratio	95% CI	*p*^t^ valor
	CS-GNB (*n* = 30)	Controls (*n* = 94)			
Demographic data					
Age in years, median (range)	66 (27–82)	62 (19–92)	1.01	0.98–1.04	0.46
Male sex, n (%)	9 (30)	46 (49)	2.24	0.93–5.39	0.07
Comorbidities^a^, n (%)					
Diabetes mellitus^b^	15 (50)	35 (37)	1.69	0.74–3.86	0.22
Renal failure^c^	8 (27)	38 (40)	0.54	0.22–1.33	0.18
Hemodialysis^d^	3 (10)	18 (19)	0.47	0.13–1.72	0.25
Chronic liver disease^e^	2 (7)	9 (10)	0.67	0.14–3.31	0.63
Immunossupressive condition^f^	6 (20)	11 (12)	1.89	0.63–5.63	0.26
Gastrointestinal disease	9 (30)	18 (19)	1.81	0.71–4.61	0.21
Geniturinary disease	3 (10)	9 (10)	1.05	0.27–4.16	0.95
Pulmonary disease	7 (23)	14 (15)	1.74	0.63–4.82	0.29
AIDS or chronic infectious disease	2 (7)	3 (3)	2.17	0.35–13.62	0.41
Surgery^g^	19 (63)	46 (49)	1.80	0.77–4.20	0.17
Infection/Colonization by CR-GNB	5 (17)	7 (7)	2.49	0.73–8.51	0.15
Nosocomial diarrhea^h^	5 (17)	6 (6)	2.93	0.83–10.42	0.10
Neutropenia^i^	1 (3)	4 (4)	0.78	0.08–7.22	0.82
Neoplasm	17 (57)	28 (30)	3.08	1.32–7.19	0.009
Infection	10 (33)	31 (33)	0.92	0.39–2.21	0.86
Prior ICU hospitalization, n (%)	9 (30)	5 (5)	7.63	2.32–25.13	0.001
Length of hospital stay (in days)					
Median (range)	15 (0–142)	10 (0–143)	1.01	0.99–1.03	0.16
ICU hospitalization reason, n (%)					
Elective or emergency surgery	10 (33)	28 (30)	1.18	0.49–2.84	0.71
Respiratory tract disease	5 (17)	14 (15)	1.14	0.38–3.49	0.82
Cardiovascular disease	1 (3)	8 (9)	0.37	0.04–3.09	0.36
Neurological disease	3 (10)	11 (12)	0.84	0.22–3.23	0.80
Gastrointestinal disease	1(3)	5 (5)	0.61	0.07–5.47	0.66
Renal pathology	1 (3)	11 (12)	0.26	0.03–2.10	0.21
Sepsis	10 (33)	30 (32)	1.07	0.45–2.56	0.89
Sepsis shock	10 (33)	27 (39)	1.24	0.51–2.99	0.63
Total SOFA score^j^ at ICU admission,					
Median (range)	6 (1–18)	6 (0–17)	1.03	0.93–1.14	0.58
SAPs 3 score^k^, median (range)	66 (27–97)	64 (29–105)	1.00	0.98–1.02	0.95
Invasive devices, n (%)					
Mechanical ventilation	18 (60)	58 (62)	0.97	0.42–2.25	0.95
Central vascular catheter	25 (83)	81 (86)	0.80	0.26–2.47	0.70
Urinary catheter	23 (77)	72 (76)	1.00	0.38–2.65	0.99
Previous use of antimicrobials, n (%)					
Aminoglycosides^l^	2 (7)	7 (7)	0.89	0.17–4.52	0.89
Cephalosporins, 3rd and 4rd generations^m^	5 (17)	16 (17)	0.98	0.32–2.93	0.96
Carbapenems^n^	5 (17)	32 (34)	0.39	0.14–1.11	0.08
Glycopeptides^o^, linezolid and tigecycline	7 (23)	35 (37)	0.51	0.19–1.30	0.17
Fluoroquinolones^p^	2 (7)	15 (16)	0.38	0.08–1.75	0.21
Metronidazole	7 (23)	13 (14)	1.90	0.68–5.31	0.22
Piperacilin-tazobactam	8 (27)	21 (22)	1.26	0.49–3.25	0.63
Polymyxins^q^	3 (10)	9 (10)	1.05	0.27–4.16	0.95
ATB with action for anaerobes^r^	14 (47)	59 (63)	0.52	0.23–1.19	0.12
Antifungal agents^s^	3 (10)	13 (14)	0.69	0.18–2.61	0.59

*Abbreviations*: *AIDS* Acquired Immunodeficiency Syndrome, *ATB* antibiotic agents, *CI* Confidence interval, *CR-GNB* Carbapenem resistant Gram-negative bacilli, *ICU* Intensive Care Unit

^a^Prior comorbidities or conditions to investigated sepsis episode

^b^Diagnosis of diabetes mellitus requiring oral or injectable hypoglycemic drug

^c^Creatinine clearance < 30 cc/min

^d^Required in the last 90 days

^e^Laboratory clinical evidence

^f^Prednisone > 10 mg for more than 50 days, corticosteroid for > 7 days or immunomodulatory agents (examples: monoclonal agents, methotrexate)

^g^In the last 30 days

^h^Nosocomial diarrhea (3 or more daily episodes of stool for 2 or more days)

^i^Granulocytes < 500 cells/mm^3^

^j^Sequential Organ Failure Assessment score

^k^Simplified Acute Physiology Score III

^l^Amikacin and gentamicin

^m^Ceftriaxone, ceftazidime and cefepime

^n^Ertapenem, imipenem-cilastatin and meropenem

^o^Daptomycin, teicoplanin and vancomycin

^p^Ciprofloxacin, levofloxacin, moxifloxacin

^q^Polymyxin B and colistin

^r^Antibacterial agents with action for anaerobes – Amoxicilin-clavulanate, ampicillin-sulbactam, piperacilin-tazobactam, clindamycin, ertapenem, imipenem, meropenem and metronidazole

^s^Amphotericin B family (standard, lipid complex or liposomal Amphotericin), echinocandins and azoles

^t^Pearson’s chi-square test or Fisher’s exact test or Mann-Whitney-Wilcoxon U test, as required, and considering statistically significant *p* < 0.05

### Definitions

Recurrent sepsis was defined as a new episode of sepsis developing after resolution of clinical and laboratory parameters of sepsis, or the recrudescence of sepsis with the evidence of new etiology by cultures during ICU stay. An adaptation of Singer et al. (2016) [21] sepsis-3 criteria was used retrospectively as follows: delta SOFA ≥2 between SOFA scores measured on two calendar days between the period of 72 h that preceded to 24 h that succeeded the date of initial blood culture, and on the ICU admission date; and qSOFA applied in patients without mechanical ventilation or sedation within 72 h before and 24 h after the date of blood culture.

Post-hoc analysis was performed by two research infectious disease physician investigators to review all clinical, radiological, and microbiological data. We reviewed the evidence of sepsis-2 [22] and − 3 [21], and of the infectious source. We classified the plausibility of infectious source as definitive, probable, possible or undetermined, according to Klouwenberg et al. (2013) criteria [23], adapted to include the updated Centers for Disease Control and Prevention definitions [24].

### Microbiological methods

Blood cultures (aerobic and anaerobic) were processed using the BD BACTEC™ system (Becton Dickinson, Sparks, MD, EUA), according to the routine of hospital microbiology laboratory. Identification and antibiotic susceptibility testing of any culture isolate were performed by VITEK®2 (BioMérieux, Hazelwood, MO, USA) system, and confirmed by disk diffusion or E-test, according to the updated recommendations of Clinical and Laboratory Standards Institute [25] and European Committee on Antimicrobial Susceptibility Testing [26], including the use of meropenem, imipenem and ertapenem for all Gram-negative bacterial species, except *Stenotrophomonas maltophilia* which is naturally resistant to carbapenems. Carbapenemase production was investigated by phenotypic tests with phenylboronic acid and ethylene diamine tetra acetic acid.

Gram-negative bacterial isolates detected in blood and other cultures were referred to Laboratório de Pesquisa em Infecção Hospitalar for microbiological confirmation by using classical and molecular biochemical methods as described in previous publications [27, 28]. The search for the following genes of carbapenemases of Amber class A (*bla*_KPC-2_), B (*bla*_SPM-1_, *bla*_NDM-1,_ *bla*_VIM_) and D (*bla*_OXA-23-like_, *bla*_OXA-48-like_ and *bla*_OXA-51-like_) was performed by using in-house multiplex polymerase chain reaction (PCR) test.

We classified the antimicrobial susceptibility profile of the strains in multi-drug (MDR), extensively-drug (XDR) and pan-drug resistant (PDR) and described as “possible” profiles whenever not all antimicrobials of all selective classes for each bacterial group or species were tested, according to Magiorakos et al. (2012) [29]. *S. maltophilia* and *Burkholderia cepacia* were considered MDR.

### Sample size and statistical analysis

Considering an alpha error of 5%, a power of 80%, a control to case ratio of 1:1, and respectively 40 and 18% exposure to carbapenem among cases and controls [19], the sample size estimated was 152 patients.

The findings were used to build a model with clinical-epidemiological factors that can be easily identified by physicians during the first moment of patient evaluation, at a time when only clinical-epidemiological parameters can guide empirical antimicrobial therapy. All variables were analyzed using SPSS® statistics v22.0 software. Categorical variables were compared using Chi-Square or Fisher’s exact test and for continuous variables, the Mann-Whitney-Wilcoxon test was used. Collinearity was investigated initially using Pearson correlation matrix and cross-tabulations between two or more variables [30]. All variables investigated as predictors were explored in univariate and multivariate logistic regression analyses using the complete data set to identify independent risk factors for CR-GNB and CS-GNB sepsis [31].

To optimize the model, we used our best knowledge not to include variables with collinearity together and give chances to those clinically meaningful. Our database has many variables that may be related to our outcome and correlated with each other. Although all variables were considered, those with small frequency and those with collinearity had to be excluded from multivariate analysis to improve the fit of the model. Possible interactions were also investigated. Using the approach described above, different models were evaluated but the best-fit model came with backward selection procedure. We also have used robust fit criteria for model comparisons (AIC and BIC) [32]. Both sides of the curve and significance level of 5% were considered in all tests.

## Results

### Study population and clinical characteristics

**Fig. 1:**
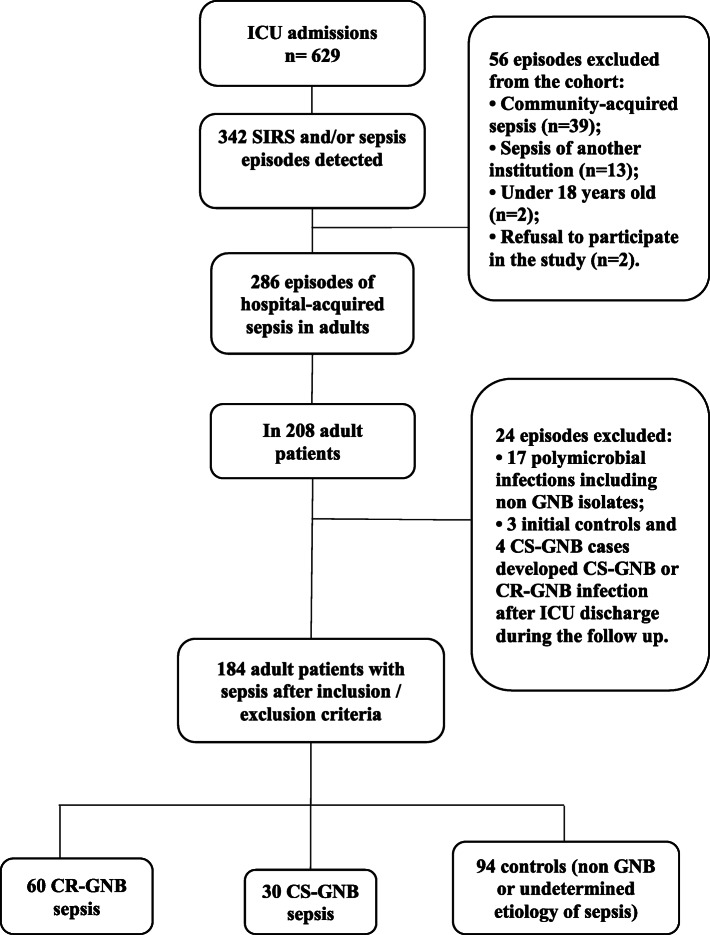
Flowchart of patients included in the study. CR-GNB carbapenem-resistant Gram-negative bacilli; CS-GNB carbapenem-susceptible Gram-negative bacilli

Among the total of 629 ICU admissions followed by 7797 patient-days, we evaluated 342 episodes of SIRS/sepsis detected (Fig. 1). After applying the exclusion criteria, we enrolled a total of 184 patients: 60 patients who acquired CR-GNB sepsis, 30 patients with CS-GNB sepsis, and each group was compared with 94 patients with undetermined (*n* = 78) or non GNB (*n* = 16) sepsis. Demographic and clinical characteristics of included patients are shown in Tables 1 and 2. Ninety-seven percent (29/30) of CS-GNB cases had severe sepsis-2, as well as 85% (51/60) of CR-GNB and 84% (79/94) of the controls. While 94% (51/54) of CR-GNB sepsis, 100% of CS-GNB cases (27/27) and 87% (74/85) of control group that could have their episode evaluable (166/184, 90%) fulfilled sepsis-3 criteria. Eighteen patients (10%, 18/184) were considered not evaluable for the modified sepsis-3 criteria for being on mechanical ventilation and sedation. Although these patients did not have delta SOFA ≥2, they had high median SOFA score at ICU admission (5.5, range 0–15) and on the date of initial blood culture collection (4, range 0–15). Septic shock was detected in 72% (43/60) of CR-GNB sepsis, 70% (21/30) of CS-GNB and 64% (60/94) of control patients with 30-day all-cause mortality rate of 50% (30/60), 40% (12/30) and 45% (42/94), respectively.

The plausibility of infectious source was categorized as definitive or probable in 89% (25/28) and 84% (54/64) of CS-GNB and CR-GNB sepsis, respectively, while the sources of infection were determined in 64% (60/94) of controls. Bacteremia was identified in 25% (46/184) of all studied patients, and 43% (20/46) of them were diagnosed as probable catheter related bloodstream infections (BSI).

CR-GNB cases were followed for the median of 47 days (range 1–351 days), while CS-GNB cases and controls for 40.5 and 37 days (range 1–71 days in both groups), respectively, during ICU stay until the end of follow-up period. The median length of ICU stays prior to the sepsis episode investigated was 10 times higher among CR-GNB cases (10 days, range 0–291) than CS-GNB cases (1-day, range 0–34) (*p* = 0.006). Half of sepsis episodes were detected at ICU admission**.** Sepsis before 48 h of ICU hospitalization occurred in 67% (20/30) of CS-GNB cases group, similar to the controls (57%, 54/94), and was statistically different among CR-GNB cases (20%, 12/60) (OR = 8.0; 95% CI 3.0–21.5; *p* < 0.001). Therefore, the majority of later sepsis was caused by CR-GNB.

**Fig. 2:**
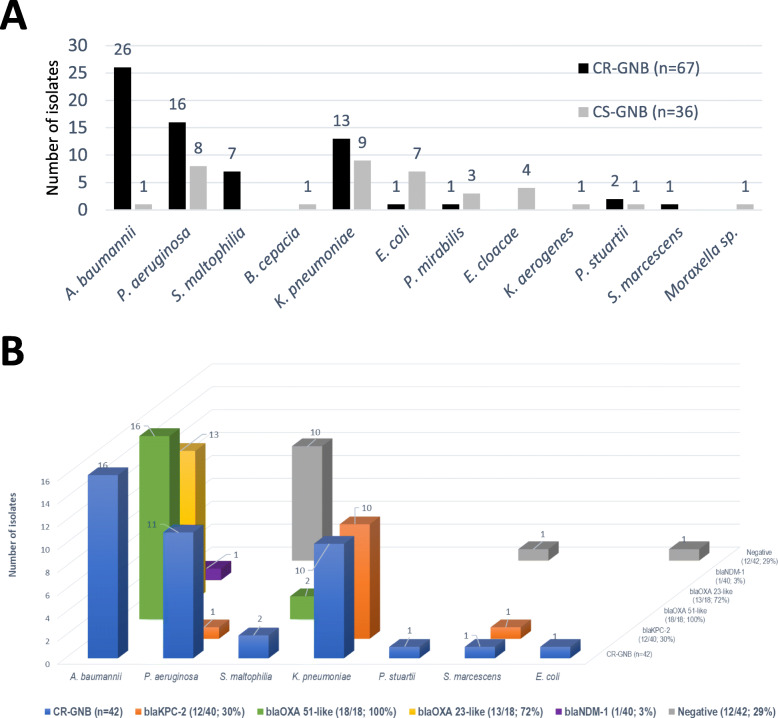
**a** Distribution of the etiological agents of sepsis by CR-GNB (*n* = 67) and CS-GNB (*n* = 36); **b** CR-GNB isolates tested (*n* = 42) and respective carbapenemase-producing genes detected (*n* = 44). CR-GNB, carbapenem resistant Gram-negative bacilli; CS-GNB, carbapenem-susceptible Gram-negative bacilli

Non-fermenting bacilli corresponded to 73% (49/67) of the etiology of CR-GNB sepsis, and *A. baumannii* accounted for 43% of the occurrences (26/60) (Fig. 2a). CR-GNB isolates were detected in tracheal aspirate or bronchoalveolar lavage (BAL) (59%, 37/63), blood (35%, 22/63), urine (3%, 2/63) and operative wound samples (3%, 2/63). The majority of isolates (83%, 45/54) had MIC ≥16 mg/ml for meropenem and/or imipenem. Two isolates of *Klebsiella pneumoniae* had MIC ≥16 mg/ml for polymyxins and 8% (4/52) of strains belonged to polymyxins naturally resistant species. CR-GNB strains causing sepsis were 89% MDR (48/54), 69% (37/54) possible extensively drug-resistant and 2% (1/54) had possible pandrug-resistant profile. The production of carbapenemases was estimated in 77% (39/51) of the isolates tested by phenotypic tests (92%, 12/13) and/or by PCR technique (74%, 35/47), since we included tested (*n* = 2) but also non-tested *S. maltophilia* isolates (*n* = 5).

Figure 2b shows the CR-GNB isolates investigated (*n* = 42) and respective carbapenemase-producing genes detected (*n* = 44). Species of the Enterobacteriaceae family (69%; 25/36) outweighed among the etiologies of CS-GNB sepsis (Fig. 2a). The isolates were recovered from blood (33%, 11/33), tracheal aspirate or BAL (30%, 10/33), operative wound samples (21%, 7/33) and urine (15%, 5/33). The susceptibility profiles of the isolates were 79% (27/34) non-MDR and 21% (7/34) classified as MDR. Polymyxin intrinsic resistance was detected in 13% (4/31) of CS-GNB isolates.

Ventilator-associated pneumonia (VAP) was the most frequent infectious source among CR-GNB sepsis (48%; 32/66) with the predominance of *A. baumannii* in 56% (18/32) of etiological agents. Among CS-GNB sepsis, respiratory tract infections (VAP, hospital-acquired pneumonia and tracheobronchitis) occurred in 37% (11/30). Intra-abdominal surgical site (SSI) plus urinary-tract (UTI) infections prevailed in CS-GNB sepsis (43%; 13/30) than CR-GNB sepsis (11%; 7/66) (*p* < 0.001). While in the control patients, excluding cases with undetermined focus (36%, 34/94), hospital-acquired pneumonia (32%, 21/66) and surgical wound (32%, 21/66) predominated as source of sepsis.

### CR-GNB septic patients versus control patients

The results of comparative univariate analysis between cases of CR-GNB sepsis versus controls are shown in Table 1. Previous infection was an important factor associated with CR-GNB sepsis (77%; 46/60) when compared to control group, with high percentage of healthcare-associated infection (57%; 26/46) and 48% (22/46) of previous sepsis at the same hospitalization period. Previous infection was predominantly of bacterial origin among CR-GNB cases (50%; 30/60) than controls (27%; 25/94) (OR = 2.74; 95% CI 1.39–5.48*; p* = 0.004). Sepsis as a reason for ICU admission occurred in 52% (31/60) of the CR-GNB cases, contrasting with only 32% (30/94) of controls (OR = 2.27; 95% CI 1.16–4.46; *p* = 0.016). Sepsis recurred during ICU stay in 65% (39/60) of CR-GNB cases and only 19% (18/94) of controls (OR = 7.71; 95% CI 3.72–16.51; *p* < 0.0001).

There was no difference between cases (77%; 46/60) versus controls (79%; 74/94) in respect to the use of antibiotics during the 3 days prior to the collection of blood culture (*p* = 0.76), including antibiotics active on GNB (70% vs. 73%; *p* = 0.64) or polymyxins (28% vs. 18%; *p* = 0.13) were used. However, there was a difference in carbapenem consumption among these groups (36/60, 60% vs. 39/94, 41%; OR = 2.11; 95% CI 1.09–4.12; *p* = 0.026).

The independent risk factors for CR-GNB sepsis are previous infection (mostly hospital-acquired bacterial infection or sepsis) (OR = 4.28; 95% CI 1.77–10.35; *p* = 0.001), previous use of mechanical ventilation (OR = 4.21; 95% CI 1.17–15.18; *p* = 0.028) and carbapenems (OR = 3.42; 95% CI 1.37–8.52; *p* = 0.008), and length of hospital stay (OR = 1.03; 95% CI 1.01–1.05; *p* = 0.007) (Table 3).

**Table 3:** Independent predictive factors associated with CR-GNB sepsis; multivariate logistic regression

Risk factors for sepsis by CR-GNB	CR-GNB cases (*n* = 60)	Control group (*n* = 94)	Odds ratio	95% CI	*p*^a^ value
Previous infection, n (%)	46 (77)	31 (33)	4.28	1.77–10.35	0.001
Mechanical ventilation, n (%)	56 (93)	58 (62)	4.21	1.17–15.18	0.028
Use of carbapenem, n (%)	48 (80)	32 (34)	3.42	1.37–8.52	0.008
Length of hospital stay (days), median (range)	26 (1–375)	10 (0–143)	1.03	1.01–1.05	0.007

*Abbreviations*: *CR-GNB* carbapenem-resistant Gram-negative bacilli, *CI* confidence interval

^a^Wald test for logistic regression, *p* significant < 0.05, accuracy of 80% for CR-GNB model

### CS-GNB septic versus control patients

Table 2 shows the comparative univariate analysis between cases of CS-GNB sepsis versus controls. Repeated episodes of sepsis occurred during ICU stay in 23% (7/30) of CS-GNB cases with no significance when compared to controls (*p* = 0.619). Regarding the use of antimicrobial agents during the 3 days prior to the initial blood culture that could interfere with its result, there was statistically significant difference between CS-GNB sepsis (53%; 16/30) and controls (79%; 74/94) (OR = 0.31; 95% CI 0.13, 0.75; *p* = 0.009). The same occurred with the use of carbapenems (3/30, 10% vs. 39/94, 41%; OR = 0.16; 95% CI 0.03–0.57; *p* = 0.0009). However, patients with CS-GNB sepsis did not receive polymyxins previously, but control patients received them (18%; 17/94).

In multivariate analysis, readmission to the ICU (OR = 6.92; 95% CI 1.72–27.78; *p* = 0.006) and prior nosocomial diarrhea (OR = 5.32; 95% CI 1.07–26.45; *p* = 0.041) were detected as independent risk factors for developing CS-GNB sepsis in the study population.

## Discussion

In this study, patients with longer length of hospital stay, previous infection, mostly hospital acquired bacterial infection, who had been previously treated with mechanical ventilation and carbapenems presented a higher risk of sepsis due to CR-GNB than control group. Whereas readmission to the ICU and prior nosocomial diarrhea were factors associated with CS-GNB sepsis in the study population.

Previous infection has been rarely reported as risk for infection/colonization or bacteremia by CR *A. baumannii* [7, 8]. In our cohort, previous infection was the most important factor for CR-GNB sepsis, mainly of bacterial origin, mostly nosocomial infection and 48% previous sepsis. Recurrent pattern of sepsis was a striking feature of our studied population. Therefore, more attention for the prevention and control of nosocomial sepsis is required for the prevention of subsequent hospital-acquired CR-GNB sepsis, especially caused by *A. baumannii*, at the same hospitalization period in ICU patients.

The studies that have evaluated risk factors for CR-GNB or CR *A. baumannii* bacteremia, SIRS or nosocomial infection have found mechanical ventilation, respiratory failure, but also VAP as risk factors in critically ill patients [7, 8, 18, 19]. In our study we did not investigate types of infection as predictive factors because we sought to investigate variables that would readily discriminate patients with increased risk, in order to be useful to guide empirical therapy in future.

Prolonged hospital stay is a classically recognized risk for hospital and ICU infection [10, 11, 14, 19, 33]. We found a 2% increase in the chances of developing sepsis by CR-GNB for every day of hospitalization. ICU readmission is also a documented risk factor for the acquisition of CS-GNB infection [10, 16]. A few studies have found diarrhea to be associated with GNB bacteremia [34–36].

As far as we know, this is the first risk factor study in CR-GNB sepsis that has considered repetitive episodes and not only bacteremia, but the broad variety of infection sources commonly observed in ICU sepsis. Studies that have investigated risk factors for infections by CR-GNB species are still rare [18, 19]. In general, the studies focus on risk factors for infections by specific species such as *K. pneumoniae* [12, 13, 16] or other members of the Enterobacteriaceae [15, 17], *P. aeruginosa* [10, 11] and *A. baumannii* [7–9], and most of them in hospital infection [9, 10, 12, 15–17] or bacteremia [8, 11, 18, 19]. However, bacteremia has been detected in less than 30% of septic cases in ICU [37, 38]. The extensive use of vascular catheter has been recognized as the most important factor contributing to BSI [4, 39, 40], while VAP has predominated as source of sepsis in ICU [38]. Therefore, although bacteremia can be considered the gold standard it represents only part of the population who were diagnosed with sepsis in ICU and is frequently associated with vascular catheter infection. In addition, several risk factor studies select the first episode of infection or bacteremia only [8–10, 13, 16–19]. The successful longitudinal follow-up of this cohort study allowed identifying patients who presented recurrent sepsis, whose diagnoses were also essential to better select cases and controls. Giving the chance of inclusion of all episodes and the variety of sepsis infections may be more reliable to better discriminate patients at higher risk in ICU.

The case-case-control design is more effective for the identification of risk factors for antimicrobial resistant pathogens, avoiding bias of exposure to the antimicrobial of interest, for not using CS-GNB sepsis as control group [41, 42]. Our study aimed to confirm exposure to carbapenems as a risk for the development of CR-GNB sepsis [11, 12, 18, 19, 43], since our control group was composed of patients with sepsis and antimicrobial treatment. This methodology allows the application of the factors found in the management of empirical therapy, which would not be possible if the control was a patient without sepsis and antimicrobial treatment of the episode investigated [42].

The design resulted in the selection of cases so efficiently that the study detected a remarkable difference in the etiologies of sepsis by CR-GNB and CS-GNB. Indeed, the CR-GNB sepsis presented a predominance of non-fermenting bacteria, mainly *A. baumannii*, which was likely related to the higher prevalence of respiratory tract infections as source of sepsis [18, 19]. Whereas Enterobacteriaceae species predominated among CS-GNB sepsis, mostly associated with respiratory tract infections, but also intra-abdominal SSI and UTI.

Evaluated sepsis episodes in cases and control patients had a good agreement when considering the adapted parameters for the diagnosis of sepsis-3 [21]. The plausibility of infectious source as definitive and probable occurred in a high proportion in all groups of cases while the source of infection was determined in the majority of controls.

Our data characterize sepsis by CS-GNB mostly as hospital-acquired infection outside the ICU, whereas sepsis by CR-GNB was mainly ICU-acquired infection. This issue corroborates with a well-known fact that ICU patients have more risk of infection by resistant bacteria [33]. In fact, CR-GNB species typify a bacterial population with a high level of resistance and few treatment options, due to the often high carbapenems MIC and some combined resistance to polymyxins. Molecular investigation of carbapenemase production shows that we have determined the risk factors for CR-GNB sepsis predominantly with this mechanism of resistance, which is commonly described and disseminated worldwide [43–47].

Main limitation of this study may be described as a single center study, which indicates caution to any generalization of our findings. Adapting management strategies to the local epidemiological data is a general recommendation for the prevention and control of hospital-acquired infection, which indicates the performance of cyclic evaluations locally. The methodology of case-control selection was one of the study’s strengths. The performance of microbiological methods and the use of antimicrobials, which may have inhibited microbial growth in clinical cultures, could have influenced the risk factors results. Other non-investigated elements may also have interfered. Phylogenetic analysis of GNB isolates would have improved our knowledge about the epidemiological context of GNB hospital-acquired sepsis.

## Conclusions

Prolonged hospitalization with the development of healthcare-associated infection, requiring mechanical ventilation and treatment with carbapenems seem to be the natural history for subsequent sepsis by carbapenemase-producing GNB in this population. The concordance with the background knowledge suggests that these factors should be evaluated further for developing and validating a risk score to identify patients at higher risk for CR-GNB sepsis in ICU. Little is known about the influence of recurrent sepsis during the same hospitalization period. Consequently, investigation of repetitive episodes of sepsis in ICU patients is warranted.

## Supplementary information

**Additional file 1 Appendix S1**. Study Form 1, with variables investigated in this study. **Appendix S2**. Study Form 2, with variables investigated in this study. **Appendix S3**. Study Form 2a, with variables investigated in this study. **Appendix S4**. STROBE Statement—Checklist of items included in this study.

## Data Availability

The datasets used and/or analysed during the current study are available from the corresponding author on reasonable request.

## Abbreviations

BAL: Broncho alveolar lavage
BSI: Bloodstream infection
CS: Carbapenem-susceptible
CR: Carbapenem-resistant
GNB: Gram-negative bacilli
ICU: Intensive care units
MIC: Minimum Inhibitory Concentration
MDR: Multidrug resistant
PCR: Polymerase chain reaction
SAPS-3: Simplified Acute Physiology Score III
SIRS: Systemic Inflammatory Response Syndrome
SSI: Surgical site infection
SOFA: Sequential Organ Failure Assessment
UTI: Urinary-tract infection
VAP: Ventilator-Associated Pneumonia
XDR: Extensively drug resistant
PDR: Pan-drug resistant
