# Supplementary material for: Predictive factors for sepsis by carbapenem resistant Gram-negative bacilli in adult critical patients in Rio de Janeiro: a case-case-control design in a prospective cohort study
Source: Antimicrob Resist Infect Control. 2020 Aug 14;9:132. doi: 10.1186/s13756-020-00791-w (PMC7426895; doi:10.1186/s13756-020-00791-w)
Supplement: Supplementary file 1 — Additional file 1 Appendix S1. Study Form 1, with variables investigated in this study. Appendix S2. Study Form 2, with variables investigated in this study. Appendix S3. Study Form 2a, with variables investigated in this study. Appendix S4. STROBE Statement—Checklist of items included in this study. [file 13756_2020_791_MOESM1_ESM.docx]

**Predictive factors for sepsis by carbapenem resistant Gram-negative bacilli in adult critical patients in Rio de Janeiro: a case-case-control design in a prospective cohort study**

**Additional file 1**

Appendix S1. Study Form 1.

Appendix S2. Study Form 2.

Appendix S3. Study Form 2a.

Appendix S4. STROBE Statement—Checklist of items included in this study

Appendix S1. Study Form 1: **PREDICTIVE FACTORS FOR SEPSIS BY CARBAPENEM RESISTANT GRAM-NEGATIVE BACILLI IN ADULT CRITICAL PATIENTS IN RIO DE JANEIRO: A CASE-CASE-CONTROL DESIGN IN A PROSPECTIVE COHORT**

**1) Patient Initials:** ______________________  **2) Record:** _____________________  **3) File:** #___________

**4) Inclusion criteria**: Patients with

4.a. SIRS: ( ) yes ( ) no;

4.b. Sepsis: ( ) yes ( ) no;

4.c. Blood cultures collected: ( ) yes ( ) no;

4.d. Antimicrobial therapy instituted, for two or more days: ( ) yes ( ) no;

**5) Exclusion criteria**:

5.a. Refuses to sign the consent form: ( ) yes ( ) no;

5.b. Patients under 18 years old: ( ) yes ( ) no;

5.c. Community-acquired sepsis: ( ) yes ( ) no;

5.d. Sepsis associated with another healthcare institution (less than 48 hours of current hospitalization and no previous hospitalization in the study hospital): ( ) yes ( ) no;

5.e. Polymicrobial sepsis (except when all agents detected are BGN or all are non-BGN): ( ) yes ( ) no;

**6) Informed consent form signed and dated**: ( )Yes ( ) No **7) Age:**______ **8)Date of birth**:____/_____/_____

**9) Sex**: ( ) Male; ( ) Female; **10) Hospital admission date:** ____/____/_____

**11) Origin**: ( ) Another Hospital; ( ) Residence; ( ) Hospital Ward (describe):_____________________

**12) Hospital admission date:**___/___/___ **13) ICU admission date:**___/___/___

**14) Date of initial blood culture collection: __/___/** ____

**15) Prior ICU admission** (in the same hospitalization): ( ) yes, ( ) no, ( ) without information

**16) Antimicrobials usage during hospitalization prior to blood culture:** ( ) yes, ( ) no (used for 2 or more days - consult the prescription):

16.a. Aminoglycosides^a^: ( ) yes ( ) no;

16.b Cephalosporins, 3^rd^ and/or 4rd generations^b^: ( ) yes ( ) no;

16.c Carbapenems^c^: ( ) yes ( ) no;

16.d Glycopeptides^d^, linezolid and/or tigecycline: ( ) yes ( ) no;

16.e. Fluoroquinolones^e^: ( ) yes ( ) no;

**Patient Initials:** ______________________  **Record:** _____________________ **File:** #___________

16.f. Metronidazole: ( ) yes ( ) no;

16.g. Piperacilin-tazobactam: ( ) yes ( ) no;

16.h. Polymyxins^f^: ( ) yes ( ) no;

16.i. ATB with action for anaerobes^g^: ( ) yes ( ) no;

16.j. Antifungal agents^h^: ( ) yes ( ) no;

^a^Amikacin and/or gentamicin; ^b^Ceftriaxone, ceftazidime and/or cefepime; ^c^Ertapenem, imipenem-cilastatin and/or meropenem; ^d^Daptomycin, teicoplanin and/or vancomycin; ^e^Ciprofloxacin, levofloxacin and/or moxifloxacin; ^f^Polymyxin B and/or colistin; ^g^Antibacterial agents with action for anaerobes – Amoxicilin-clavulanate, ampicillin-sulbactam, piperacilin-tazobactam, clindamycin, ertapenem, imipenem, meropenem and/or metronidazole; ^h^Amphotericin B family (standard, lipid complex and/or liposomal Amphotericin), echinocandins and/or azoles;

**17) Comorbidities** (at admission to the ICU and prior to initial blood culture collection) – Register in parenthesis Y- yes, N- no, WI-without information:

17.a. ( ) Diabetes mellitus (diagnosis of diabetes requiring oral or injectable hypoglycemic drugs) (describe):_____;

17.b. ( ) Renal failure (creatinine clearance < 30cc/min) (describe):_______________________________________;

17.c. ( ) Hemodialysis (required in the last 90 days) (describe):__________________________________________;

17.d. ( ) Chronic liver disease (laboratory clinical evidence) (describe):____________________________________;

17.e. ( ) Immunosuppressive condition (prednisone > 10 mg for more than 50 days, corticosteroid > 40mg for more than 7 days or immunomodulatory agents (examples: monoclonal agents, methotrexate) (describe)_______________;

17.f. ( ) Gastrointestinal disease (describe):__________________________________________________________;

17.g. ( ) Genitourinary disease (describe): ___________________________________________________________;

17.h. ( ) Cystic fibrosis or Pulmonary disease (describe): _______________________________________________;

17.i. ( ) AIDS or chronic infectious disease (describe): _________________________________________________;

17.j. ( ) Surgery (in the last 30 days, describe): _________________________________________________________;

17.k. ( ) Infection/Colonization by CR-GNB (in the last 3 months, describe) (consult HICC Form): ________________;

17.l. ( ) Nosocomial diarrhea (3 or more daily stool for 2 or more days); Total duration (days);____________________;

17.m. ( ) Neutropenia (granulocytes < 500 cell/mm^3^) - Total duration (days):_________________________________; 17.n. ( ) Neoplasm (describe): _____________________________________________________________________;

17.o. ( ) Infection (describe etiology and type of infection):_______________________________________________;

**Patient Initials:** ______________________  **Record:** _____________________ **File:** #___________

**18) ICU hospitalization reason** (describe and mark all possibilities): Register in parenthesis Y-yes, N-no, WI-without information: ( ) Elective or emergency surgery; ( ) Respiratory tract disease; ( ) Cardiovascular disease; ( ) Neurological disease; ( ) Gastrointestinal disease; ( ) Renal pathology; ( ) Sepsis; ( ) Sepsis shock. Describe:_______________________________________________________________________________________

**19) Invasive devices** (prior to ICU admission to the date of initial blood culture collection):

19.a. Mechanical ventilation: ( ) yes, ( ) no;

19.b. Central vascular catheter: ( ) yes, ( ) no;

19.c. Urinary catheter: ( ) yes, ( ) no;

**20) SIRS within 24 hours prior to the initial blood culture collection:** Register in parenthesis Y-yes, N-no **( ): start date: ___/ __/ ___** [presence of two or more (to circle): > 38°C or < 36°C; > 90 bpm; > 20 ppm or PaCO_2_: < 32 mmHg; leukocytes: >12,000 cell/μL or <4,000 cell/μL or ≥ 10% of rods].

**21) SOFA score** (at ICU admission) as Table 1 below: A:_____;B:_____;C:_____;D:____;E:____F:____;Total:______

**22) SOFA score** (at initial blood culture date): A:____;B:____;C:____;D:____; E:_____;F:_____;Total: ________

**23) SOFA score (**applied retrospectively 24 to 72 hours prior initial blood culture date)**:**A:____;B:____;C:____;D:____; E:____;F:____;Total: ____

**24) Delta SOFA ≥ 2 points** (compared to initial or previous SOFA): ( ) yes, ( ) no;

**25)** **Mechanical ventilation** (at initial blood culture date or until 72 hours earlier): ( ) Yes, ( ) No, If not, answer qSOFA in parenthesis yes, no, without information ( ) **26) Quick SOFA** (presence of 2 or 3 of the following at 24 to 72 hours prior ) (to circle): Consciousness level change (Glasgow Scale <15), Respiratory rate ≥ 22bpm and systolic BP ≤100 mmHg.

**27)   Saps 3 Points** (as in ICU system): **________**

**Patient initials: _________________ Record:______________________ # File:_______________________**

**Table 1: SOFA score (to guide answers to questions 21 to 24 above)**

| **SOFA score*** | **1** | **2** | | **3** | **4** |
| --- | --- | --- | --- | --- | --- |
| **Pulmonar (A)** *PaO2/FiO2, mmHg*** | < 400 | < 300 | | < 200, with respiratory support | < 100, with respiratory support |
| **Coagulation (B)** *Platelets x 10^3^/mm^3^* | < 150 | < 100 | | < 50 | < 20 |
| **Hepatic (C)** *Bilirubin, mg/dL* | 1.2 – 1.9 | 2.0 – 5.9 | | 6.0 – 11.9 | >12.0 |
| **Cardiovascular (D)** *Hypotension* | MAP <70mmHg | Dopamine ≤ 5 or dobutamine any dose *** | | Dopamine > 5 or adrenaline ≤ 0.1 or noredrenaline ≤ 0.1 | Dopamine > 15 ou adrenaline > 0.1 or noredrenaline > 0.1 |
| **CNS (E)** *Glasgow coma scale* | 13 – 14 | 10 – 12 | | 6 – 9 | < 6 |
| **Renal (F)** *Creatinine, mg/dL or urinary volume* | 1.2 – 1.9 | 2.0 – 3.4 | | 3.5 – 4.9 ou < 500mL/dia | > 5.0 ou < 200mL/dia |
| * Vincent JL, Moreno R, Takala J, et al. The SOFA (Sepsis-related Organ Failure Assessment) score to describe organ dysfunction/failure: on behalf of the Working Group on Sepsis-Related Problems of the European Society of Intensive Care Medicine. Intensive Care Med. 1996;22:707–10; ** If arterial blood gas analysis is not available, use SatO_2_ / FiO_2_ as pulmonary parameter according to Table 2. *** Adrenergic agents administered for at least 1 hour. Doses given in μg/kg-min. | | | | | |
| **Table 2 – Pulmonary Parameter for SOFA Score for cases of unavailable arterial blood gas*** | | | | | |
| **SOFA score** | **1** | | **2** | **3** | **4** |
| **Pulmonar** *SatO2/FiO2* | **221.2 –302.3** | | **142.3 – 221.2** | **67.0 – 142.3** | **<67.0** |

***** Pandharipande PP, Shintani AK, Hagerman HE, St Jacques PJ, Rice TW, Sanders NW, et al. Derivation and validation of Spo2/Fio2 ratio to impute for Pao2/Fio2 ratio in the respiratory component of the Sequential Organ Failure Assessment score**.**Crit Care Med. 2009; 37:1317–21.

**28) Date of form completion:** ___/___/___; 29**) Researcher Initials: _**________

Appendix S2. Study Form 2: **PREDICTIVE FACTORS FOR SEPSIS BY CARBAPENEM RESISTANT GRAM-NEGATIVE BACILLI IN ADULT CRITICAL PATIENTS IN RIO DE JANEIRO: A CASE-CASE-CONTROL DESIGN IN A PROSPECTIVE COHORT**

**1) Patient initials: _________________ 2) Record:____________________ 3)# File:________________________**

**4) Diagnosis** (24 hours before or after the initial blood culture, answer in the parenthesis yes, no)**:**

4.a. ( ) Sepsis (infection proved or not with two or more general or inflammatory variables:___/___/___;

4.b. ( ) Documented sepsis (microbiologically proven infection with 2 or more general or inflammatory variables):__/__/__

4.c. ( ) Severe sepsis (sepsis with at least one organ dysfunction):___/___/___

4.d. ( ) Septic shock (sepsis with persistent hypotension that does not respond to volume replacement and requires inotropes or vasopressors):___/___/___ Lactate >2mmol (retrospectively collected):__/___/___:_____(describe highest lactate)

General Variables (circle all that apply): Fever >38°C or Hypothermia <36°C; >90 bpm; >20 bpm; Altered level of consciousness; Significant edema or positive fluid balance (>20 ml/kg within 24 h); Hyperglycemia (glucose >140 mg/dl) without diabetes.

Inflammatory Variables (circle all that apply): leukocytes: >12,000 cell/μL or <4,000 cell/μL or ≥10% of rods; CRP >2 standard deviations above normal value; Procalcitonin >2 standard deviations above normal.

Hemodynamic Variables (circle all that apply): Systolic blood pressure <89 mmHg, Mean blood pressure <70 mmHg or Systolic blood pressure decrease >40 mmHg.

Organic Dysfunction (circle all that apply): Arterial hypoxemia (PaO_2_/FiO_2_ <300), Acute oliguria (urinary output <0.5 ml/kg/h for at least 2h despite adequate fluid resuscitation); Increased creatinine >0.5 mg/dl; Coagulation disorder (INR >1.5 or PTT >60s); Ileus (lack of intestinal peristalsis); thrombocytopenia (platelets <100,000 microL); Hyperbilirubinemia (>4 mg/dl).

Tissue perfusion variable (circle all that apply): Hyperlactatemia (arterial lactate >1 mmol/l); decreased capillary filling or livedo reticularis.

**5) Type of infection source that determined sepsis** (mark all that apply, answer in the parenthesis yes, no, according to adapted Klouwenberg *et al.* (2013) criteria [22])

5.a. ( ) Sepsis undetermined focus __________________________________________________________________

5.b. ( ) Vascular catheter infection, type, location, and date of catheter removal (inform if not removed):___________

**Patient initials:_________________ 2) Record:______________________3) # File:_______________________**

5.c. ( ) Ventilator-associated pneumonia _____________________________________________________________

5.d. ( ) Hospital-acquired pneumonia________________________________________________________________

5.e. ( ) Meningitis,_______________________________________________________________________________

5.f. ( ) Endocarditis______________________________________________________________________________

5.g. ( ) Soft tissue infection________________________________________________________________________

5.h. ( ) Osteomyelitis_____________________________________________________________________________

5.i. ( ) Surgical site infection:______________________________________________________________________
5.j. ( ) Other infection:____________________________________________________________________________

5.k. ( ) Catheter-related urinary tract infection; type and date of device removal (not removed):__________________

5.l. ( ) Non-catheter-associated Urinary Tract Infection:_________________________________________________

5.m. ( ) Intra-abdominal infection (intraperitoneal, retroperitoneal, intraparenchymal, to circle):_________________

5.n. ( ) Other biofilm associated infection: ( ) chronic UTI, ( ) chronic infected decubitus ulcer, ( ) chronic sinusitis, ( ) prosthetic infection, ( ) Pneumonia in cystic fibrosis, ( ) other (describe) _______________________

**6) Signs and symptoms of infection:**

| **Event** | Date | A) Started or maintained Antimicrobial for sepsis^1^ treatment | B) Fever or hypothermia^1^**^,2^** | C) Leukocytosis or leukopenia**^1,2^** | D) Signs and symptoms at primary infection site**^1,2^** | E) Other cultures collected on that day were positive**^1^** | F) Microbiological, surgical and / or imaging evidence of infection (to circle)**^1,2^** | G) Microbiological, surgical and / or imaging evidence of infectious complication (to circle)**^1,2^** |
| --- | --- | --- | --- | --- | --- | --- | --- | --- |
| Within 24 hours of initial Blood culture date |  |  |  |  |  |  |  |  |

**^1^**Yes, No, Without information, Not applicable (NA); ^2^Describe evidence [evidence of radiological imaging with report ( )yes, ( )no, ( ) without information_________________________________________________________

**7) Concurrent infections by laboratory confirmed etiology (**at date of initial blood culture)**:**

( ) Bacterial, ( )Viral, ( ) Fungal; Start date: ___/____/____ Describe:__________________________________

**Patient initials: _________________ Record:______________________ # File:_______________________**

**8)** **Antimicrobial treatment used prior to the date of blood collection** (used up to 3 days prior to the date of blood collection, yes ( ), no ( ), without information ( )

**9) All antimicrobial used** up to 3 days prior to the date of blood collection:

| **Antimicrobial** | **Start date** | **End date** |
| --- | --- | --- |
|  | **** |  |
|  |  |  |
|  | **** |  |
|  |  |  |
|  | **** |  |
|  |  |  |
|  | **** |  |
|  |  |  |
|  |  |  |

Also follow all antimicrobial usage until 30 days post end of treatment, for two or more days.

**10) End of Antimicrobial Treatment of current episode** (the last date of antimicrobial suspension)**:**____/____/____

**11)** **End of follow-up** (30 days after the end of antimicrobial treatment):____/____/____

**12) Clinical support withdraw:** ___/___/___ ; **13) ICU discharge**:___/____/___;

**14) Death**: ___/____/____ , ( ) at ICU, ( ) post-discharge ICU and during hospitalization (answer with yes or no in the parenthesis)

**15) Date of form completion:** ___/___/___; **16) Researcher Initials: _**________

Appendix S3. Study Form 2a: **PREDICTIVE FACTORS FOR SEPSIS BY CARBAPENEM RESISTANT GRAM-NEGATIVE BACILLI IN ADULT CRITICAL PATIENTS IN RIO DE JANEIRO: A CASE-CASE-CONTROL DESIGN IN A PROSPECTIVE COHORT**

**1) Patient initials: _________________ 2) Record:____________________ 3)# File:________________________**

**4) Etiology of sepsis** (describe or undetermined, according to adapted Klouwenberg *et al.* (2013) criteria) [22], follow all microbiology exams until 30 days post the end of antimicrobial therapy:_____________________________

**4.a. Culture 1** – date:___/___/___ (describe isolate(s), clinical material and phenotypic, according Magiorakos et al. 2012) [28]: Answer yes, no, or not applicable in the parenthesis below:

1^st^ isolate: __________________________( ) NON-MDR; ( )MDR; ( ) XDR; ( ) PDR ( ) carbapenem resistance

2^st^ isolate: __________________________( ) NON-MDR; ( )MDR; ( ) XDR; ( ) PDR ( ) carbapenem resistance

3^st^ isolate: __________________________( ) NON-MDR; ( )MDR; ( ) XDR; ( ) PDR ( ) carbapenem resistance

**4. b.** **Culture 2** – date:____/___/___(describe isolate(s), clinical material and phenotypic, according Magiorakos et al. 2012) [28]: Answer yes, no, or not applicable in the parenthesis below:

1^st^ isolate: __________________________( ) NON-MDR; ( )MDR; ( ) XDR; ( ) PDR ( ) carbapenem resistance

2^st^ isolate: __________________________( ) NON-MDR; ( )MDR; ( ) XDR; ( ) PDR ( ) carbapenem resistance

3^st^ isolate: __________________________( ) NON-MDR; ( )MDR; ( ) XDR; ( ) PDR ( ) carbapenem resistance

**4. c.** **Culture 3** – date: ____/____/____ (describe isolate(s), clinical material and phenotypic, according Magiorakos et al. 2012) [28]: Answer yes, no, or not applicable in the parenthesis below:

1^st^ isolate: __________________________( ) NON-MDR; ( )MDR; ( ) XDR; ( ) PDR ( ) carbapenem resistance

2^st^ isolate: __________________________( ) NON-MDR; ( )MDR; ( ) XDR; ( ) PDR ( ) carbapenem resistance

3^st^ isolate: __________________________( ) NON-MDR; ( )MDR; ( ) XDR; ( ) PDR ( ) carbapenem resistance

**5) Comments:__________________________________________________________________________________ ____________________________________________________________________________________________________________________________________________________________________________________________________________________________________________________________________________________________________________________________________________________________________________________________****6) Date of form completion:** ___/___/___; 7**) Researcher Initials: _**________

Appendix S4. **STROBE Statement—Checklist of items included in this study**

|  | Item No | Recommendation | Page No |
| --- | --- | --- | --- |
| **Title and abstract** | 1 | (*a*) Indicate the study’s design with a commonly used term in the title or the abstract | Page 1 – Title, Lines 1 to 3 |
|  |  | (*b*) Provide in the abstract an informative and balanced summary of what was done and what was found | Pages 3 and 4, Abstract, Lines 47 to 72 |
| Introduction | | | |
| Background/rationale | 2 | Explain the scientific background and rationale for the investigation being reported | Page 4, Lines 78 to 93 |
| Objectives | 3 | State specific objectives, including any prespecified hypotheses | Page 4, Lines 90 to 93 |
| Methods | | | |
| Study design | 4 | Present key elements of study design early in the paper | Page 5, lines 95 to 98 |
| Setting | 5 | Describe the setting, locations, and relevant dates, including periods of recruitment, exposure, follow-up, and data collection | Pages 5 and 6, Lines 95 to 129 |
| Participants | 6 | (*a*) Give the eligibility criteria, and the sources and methods of selection of participants. Describe methods of follow-up | Pages 5 and 6, Lines 103 to 117 |
|  |  | (*b*) For matched studies, give matching criteria and number of exposed and unexposed | Not applicable, no matching |
| Variables | 7 | Clearly define all outcomes, exposures, predictors, potential confounders, and effect modifiers. Give diagnostic criteria, if applicable. | Pages 5, 6 and 7, Lines 107 to 112, 118 to 123 and 127 to 143 |
| Data sources/ measurement | 8* | For each variable of interest, give sources of data and details of methods of assessment (measurement). Describe comparability of assessment methods if there is more than one group | Page 6, Lines 118 to 129 |
| Bias | 9 | Describe any efforts to address potential sources of bias | Pages 5 and 6, Lines 111 to 112, and 127 to 129 |
| Study size | 10 | Explain how the study size was arrived at | Page 8, Lines 166 to 169 |
| Quantitative variables | 11 | Explain how quantitative variables were handled in the analyses. If applicable, describe which groupings were chosen and why | Page 8 and 9, Lines 173 to 187 |
| Statistical methods | 12 | (*a*) Describe all statistical methods, including those used to control for confounding | Page 8 and 9, Lines 173 to 187 |
|  |  | (*b*) Describe any methods used to examine subgroups and interactions | Lines 173 to 187 |
|  |  | (*c*) Explain how missing data were addressed | Lines 176 to 178 |
|  |  | (*d*) If applicable, explain how loss to follow-up was addressed | No loss of follow up |
|  |  | (*e*) Describe any sensitivity analyses | - |
| Results | | |  |
| Participants | 13* | (a) Report numbers of individuals at each stage of study—eg numbers potentially eligible, examined for eligibility, confirmed eligible, included in the study, completing follow-up, and analysed | Page 9, Lines 190 to 196, and Figure 1 |
|  |  | (b) Give reasons for non-participation at each stage | Figure 1 |
|  |  | (c) Consider use of a flow diagram | Figure 1 |
| Descriptive data | 14* | (a) Give characteristics of study participants (eg demographic, clinical, social) and information on exposures and potential confounders | Tables 1 and 2 |
|  |  | (b) Indicate number of participants with missing data for each variable of interest | Tables 1 and 2 |
|  |  | (c) Summarise follow-up time (eg, average and total amount) | Page 10, Lines 210 to 214 |
| Outcome data | 15* | Report numbers of outcome events or summary measures over time | - |

| Main results | 16 | (*a*) Give unadjusted estimates and, if applicable, confounder-adjusted estimates and their precision (eg, 95% confidence interval). Make clear which confounders were adjusted for and why they were included | Tables 1, 2 and 3, Pages 11 to 13, Lines 246 to 277 |
| --- | --- | --- | --- |
|  |  | (*b*) Report category boundaries when continuous variables were categorized | - |
|  |  | (*c*) If relevant, consider translating estimates of relative risk into absolute risk for a meaningful time period | - |
| Other analyses | 17 | Report other analyses done—eg analyses of subgroups and interactions, and sensitivity analyses | - |
| Discussion | | | |
| Key results | 18 | Summarise key results with reference to study objectives | Pages 13 and 16; Lines 280 to 284 and 356 to 358 |
| Limitations | 19 | Discuss limitations of the study, taking into account sources of potential bias or imprecision. Discuss both direction and magnitude of any potential bias | Page 16, Lines 345 to 353 |
| Interpretation | 20 | Give a cautious overall interpretation of results considering objectives, limitations, multiplicity of analyses, results from similar studies, and other relevant evidence | Page 16, Lines 356 to 362 |
| Generalisability | 21 | Discuss the generalisability (external validity) of the study results | Page 16, Line 345 to 346 |
| Other information | | | |
| Funding | 22 | Give the source of funding and the role of the funders for the present study and, if applicable, for the original study on which the present article is based | Page 17, Lines 381 to 383 |

*Give information separately for exposed and unexposed groups.

**Note:** An Explanation and Elaboration article discusses each checklist item and gives methodological background and published examples of transparent reporting. The STROBE checklist is best used in conjunction with this article (freely available on the Web sites of PLoS Medicine at http://www.plosmedicine.org/, Annals of Internal Medicine at http://www.annals.org/, and Epidemiology at http://www.epidem.com/). Information on the STROBE Initiative is available at http://www.strobe-statement.org.
